# The attributes of the images representing the SARS-CoV-2 coronavirus affect people’s perception of the virus

**DOI:** 10.1371/journal.pone.0253738

**Published:** 2021-08-25

**Authors:** Celia Andreu-Sánchez, Miguel Ángel Martín-Pascual

**Affiliations:** 1 Neuro-Com Research Group, Audio-visual Communication and Advertising Department, Universitat Autònoma de Barcelona, Barcelona, Spain; 2 Serra Húnter Fellow; 3 Technological Innovation, Instituto Radio Televisión Española (IRTVE); University of Haifa, ISRAEL

## Abstract

**Background:**

The recent COVID-19 pandemic has seen an explosion of coronavirus-related information. In many cases, this information was supported by images representing the SARS-CoV-2.

**Aim:**

To evaluate how attributes of images representing the SARS-CoV-2 coronavirus that were used in the initial phase of the coronavirus crisis in 2020 influenced the public’s perceptions.

**Methods:**

We have carried out an in-depth survey using 46 coronavirus images, asking individuals how beautiful, scientific, realistic, infectious, scary and didactic they appeared to be.

**Results:**

We collected 91,908 responses, obtaining 15,315 associations for each category. While the reference image of SARS-CoV-2 used in the media is a three-dimensional, colour, illustration, we found that illustrations of the coronavirus were perceived as beautiful but not very realistic, scientific or didactic. By contrast, black and white coronavirus images are thought to be the opposite. The beauty of coronavirus images was negatively correlated with the perception of scientific realism and didactic value.

**Conclusion:**

Given these effects and the consequences on the individual’s perception, it is important to evaluate the influence that different images of SARS-CoV-2 may have on the population.

## Introduction

The COVID-19 pandemic provoked by the SARS-CoV-2 coronavirus has increased the delivery of scientific information to the public at large, adding pressure on media channels to ensure that the information provided is correctly interpreted [1]. While the lockdown of Wuhan City seemed to slow the international spread of COVID-19 and although the epidemic was expected to be short-lived [2], the declaration of a state of emergency in North America to halt COVID-19 greatly increased citizen’s desires for information [3]. The European Broadcasting Union (EBU) indicates that Public Media Services (PSM) evening news nearly doubled its average audience during the peak of the COVID-19 crisis in the most affected areas and on the days where key announcements were made [4]. On average, there was a 14% increase in daily views of the PSM’s evening news and visits to PSM daily online news websites increased by up to 2.7 [4].

**Fig 1:**
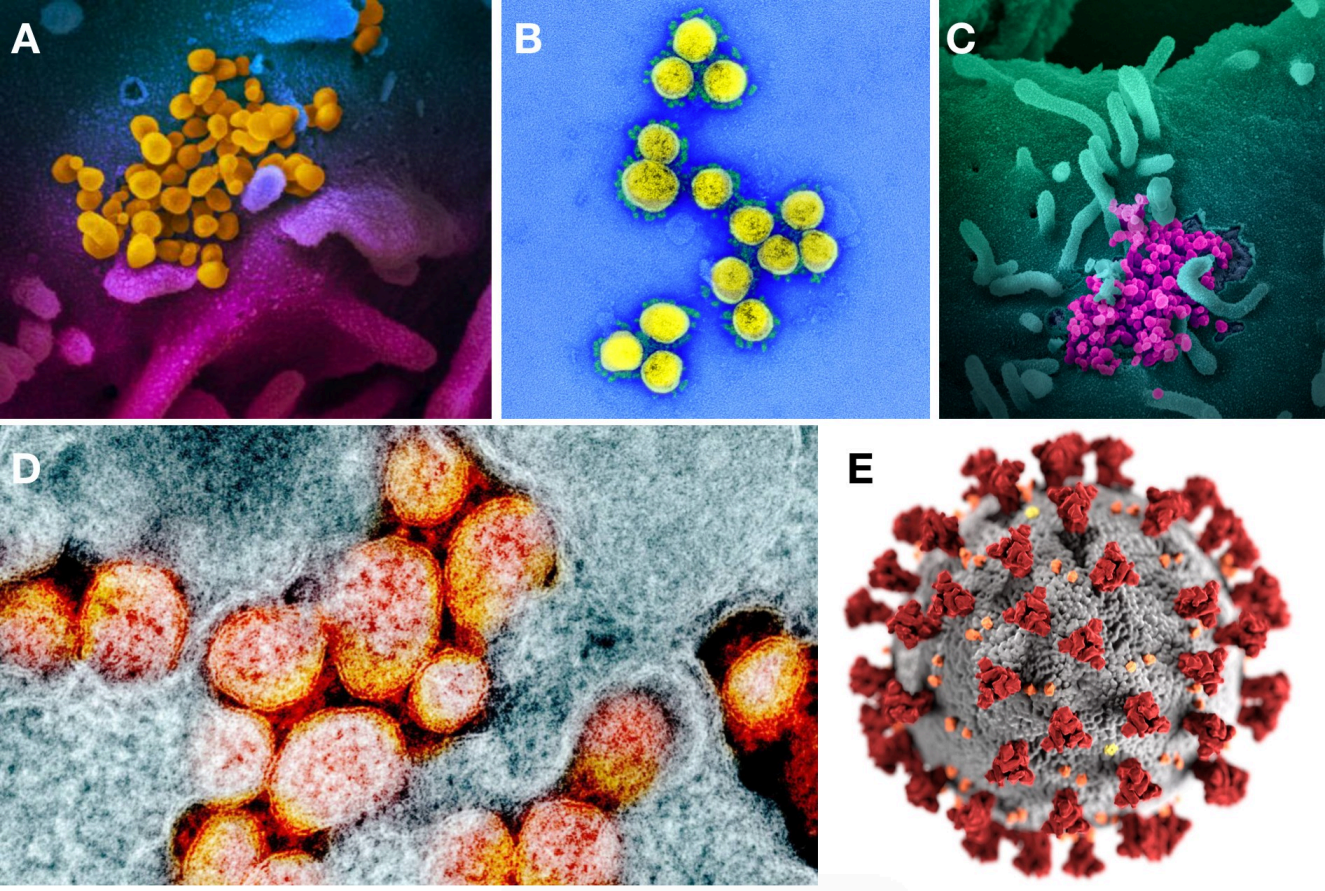
Some of the first images of SARS-CoV-2. A, B, C, D: Early real images with false colour of the coronavirus published on February 13th, 2020 by the NIAID. E: First public-domain 3D model of the coronavirus from the CDC published on January 30th, 2020. Credits: A, B, C, D, NIAID. E, Public Health Image Library (PHIL), by Alissa Eckert and Dan Higgins.

In many cases, the information related to this crisis was supported by images representing the coronavirus [5]. The first real image of the coronavirus, in black and white, was published by the Chinese Center for Disease Control and Prevention (CCDC) on January 24^th^, 2020 [6]. Subsequently, the Rocky Mountain Laboratories (RML) at the National Institute of Allergy and Infectious Diseases (NIAID) revealed new, higher resolution images of the coronavirus on February 13th, 2020, enhanced in false colour [7]. On March 5th, 2020, high resolution structural and morphological cryogenic electron microscopy (cryoEM) representations of the coronavirus in black and white were also published [8]. In addition, on January 30th, 2020, the Center for Disease Control and Prevention in the USA (CDC) published the first public-domain model of the SARS-CoV-2 designed in 3D, a reasonably scientific reconstruction of the morphology of the coronavirus [9] (Fig 1).

**Fig 2:**
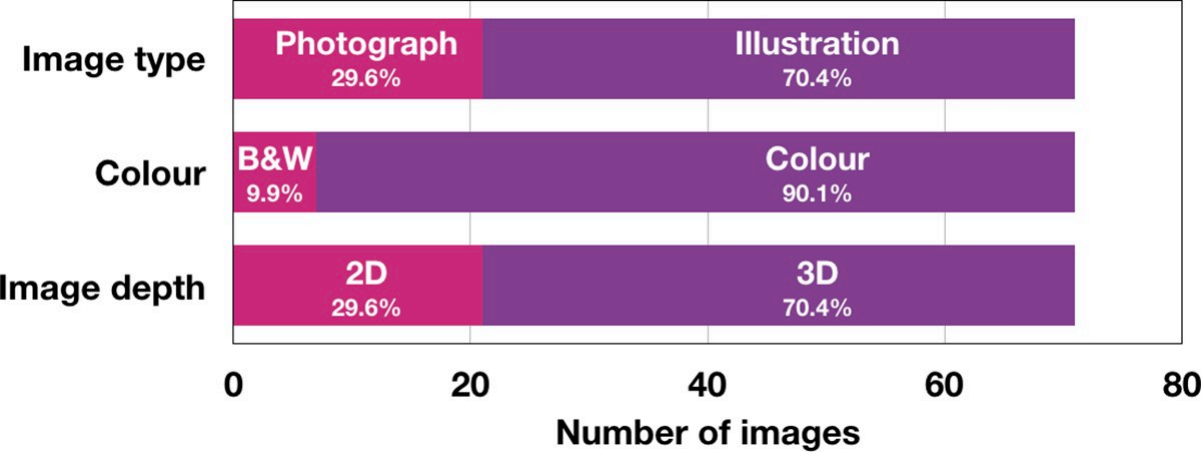
Attributes and format of SARS-CoV-2 coronavirus images. These images (N = 71) were available online at the start of the COVID-19 pandemic [5]: 29.6% of the images were photographs and 70.4% were illustrations; 9.9% were in black and white and 90.1% were in colour; and 29.6% were in two dimensions, while 70.4% were in three dimensions.

However, when we recently analysed images related to the SARS-CoV-2 coronavirus that causes COVID-19 available at the start of the pandemic, we found that the media most often used a retouched, three-dimensional, colour illustration of the coronavirus rather than a real image (Fig 2) [5]. We have also seen that many images used to support COVID-19 information are representations of other viruses and not that of SARS-CoV-2, and they are frequently enhanced by media design services to adjust the colour and other attributes of the coronavirus to their brand image [5]. Indeed, false images or imaginary illustrations of the COVID-19 coronavirus predominate in all sources of information, except in encyclopaedias or scientific articles [5].

The rigor and quality of the information in scientific communication is vital [10]. Not only should fake news be avoided but also, fake scientific communications [11]. Indeed, news stories about treatments and procedures may have a profound impact on the public at large [12], and importantly the consumers and providers of healthcare [13]. An important part of the credibility of scientific information comes from the appraisal of the images used to support it [14]. However, it is not fully clear how images of SARS-CoV-2 affect an individual’s emotions and their perception of the virus. Indeed, it remains unclear if such images change our social behaviour regarding the COVID-19 pandemic.

Visual factors in images affect our emotional processing [15] and while in this pandemic there has been a proliferation of studies analysing the public’s emotional status [16–18], we are unaware of any studies that have assessed the impact that images of may have had on this. Here, we set out to evaluate how attributes of the images representing the SARS-CoV-2 coronavirus used in the initial phase of the coronavirus crisis of 2020 may influence their audience’s emotions and perceptions. By looking at the perceptions provoked by different types of images representing the virus, we aimed to explore which formal characteristics have an important role when assessing specific categories of interest.

## Materials and methods

### Study design

We designed a study to assess how the different attributes of images representing the SARS-CoV-2 coronavirus affect people’s perception of the virus. The population studied were people affected by the COVID-19 crisis who received information about it. Since almost everybody was (more or less) affected and received information, it is difficult to make a more concrete definition of our population. To avoid answers from those who did not followed our criteria, we informed potential participants that our goal was to find out how formal elements of images representing SARS-CoV-2 in the media affected people’s perception of the virus, expecting that those who did not understand the topic would not participate in the survey. The present study is part of a wider project which included other information about: (1) how the participants remained informed during the pandemic; (2) sports activities undertaken during the pandemic; and (3) the participant’s feelings and emotions assessed using a Positive and Negative Affect Schedule (PANAS).

### Participants

We anticipated obtaining a sample of 300 participants, although we did not calculate a sample size because at the time the survey was planned (early March 2020), it was very difficult (if not impossible) to determine what our population size might be. At that time, it was difficult to exactly determine who was being affected by and receiving information about the COVID-19 crisis. Almost everyone on the planet was aware of the pandemic at some point, yet there was no clear information as to how they were affected by the crisis at that time. The participants did not receive any compensation for answering an in-depth questionnaire. They were recruited through different online channels, including: newsletters related to media communications and professors; banners on the University Autònoma de Barcelona (UAB) website and on the Spanish Public Television (RTVE) website; and posts in Facebook groups about science and COVID-19. The wording of these adverts indicated that we were carrying out a study to analyse the perceptions and emotions of citizens evoked by the images used to illustrate the SARS-CoV-2 coronavirus. They also stated that the goal was to draw conclusions that would help improve informative rigor, the quality of scientific dissemination, and provide communication professionals with tools to address the emotional effects of the COVID-19 pandemic (and other future crisis of a similar nature) on society. These adverts and banners on the web pages were linked to the questionnaire.

### Ethical approval

Ethical approval was obtained from the Ethics Committee on Animal and Human Experimentation (CEEAH) at the UAB (Spain: reference number CEEAH 5109). Due to the confinement associated with the SARS-CoV-2 pandemic, this study was carried out online and as such participants could not provide their signed informed consent. They were presented with the consent form before starting the online questionnaire and if they chose to offer their consent, they pressed the Questionnaire’s start button: “By pressing the NEXT button and answering the survey questions below, I confirm that I am over 18 years old and that I offer my informed consent to participate in this study”. This formula was approved by the CEEAH at the UAB and thus, the participants gave their informed consent by simply answering the questionnaire. The participation was voluntary, the data obtained was anonymous, and the CEEAH approval of the protocols indicated that they were in accordance with the tenets of the Helsinki Declaration and other relevant international codes and guidelines.

### Questionnaire

The online questionnaire was open to each visitor of the site, i.e.: it was not password protected and registration was not needed. The contact mode was exclusively via Internet. The questionnaire was distributed between April 3^rd^ and May 7^th^ 2020, it had four parts and it required approximately 35–45 minutes to complete. The first part presented information about the study and requested the subject’s informed consent. By pressing the NEXT button to go to the second part, the participants were informed that they accepted the informed consent. The second part required the participants to introduce personal data, including their age range or educational level, while their anonymity was maintained. The third part included questions about the media the participants used to obtain information during the pandemic, the sports and activities they practiced during the pandemic or period of social isolation, and a PANAS as complementary information (not for the purpose of this article). After again pressing the NEXT button at the end of the page, the participants could start the last part of the questionnaire. In this part of the questionnaire, 46 selected images (see “stimuli” section below) were presented to the participants in a randomized order and they were required to score the beauty, the scientific quality, the realism, the level of infectivity, the level of fear, and the didactic nature that the image of the coronavirus represented to them on a scale of 1 to 5 (1 = very mild, 2 = a little, 3 = moderately, 4 = quite strongly, 5 = extremely). Hence, the participants were asked to respond to questions related to these categories (e.g., do you think the coronavirus in this image is realistic?), although they were unaware of the specific attributes of the images that we were interested in (photograph, illustration, black and white, colour, 2D and 3D representation). Prior to distributing the questionnaire among the participants, a pilot study was carried out on 10 selected subjects (colleagues) who offered their opinion on the questionnaire to the researchers. Some changes were made on the basis of this and a second pilot study was carried out on the same 10 subjects. It was mandatory to answer all the questions on the questionnaire and if participants skipped questions, a note appeared that the questionnaire could not be submitted until it was complete. Participants were able to navigate back and forth through the questionnaire. The questionnaire was created in two versions using Google Forms, Spanish and English, and the flow of the questionnaire was checked prior to distribution. While checking Google Forms, we detected a bug whereby duplicate responses could be presented unintentionally should a user submit a response and fail to close the page, and the page reloaded for some reason. We believe this is more likely to happen on mobile devices given the way browsers reload each time they are called and as a result, the responses of a participant using such a device are sent again. To ensure this bug did not introduce any important errors, we checked for any duplicated responses once the survey was closed and none were found. The data obtained was automatically registered in a private database.

### Stimuli

To obtain the stimuli (images), an internet image search was carried out using various search engines between March 10^th^ and March 16^th^, 2020. A total of 71 different images representing coronavirus were collected using the following search terms: “SARS-CoV-2” and “COVID-19”. We selected images from non-repository sources, such as web diaries, information pages, health and medical blogs, information pages on the pandemic, pages of official or public institutions, foundations, and some photographs from scientific articles that characterized the SARS-CoV-2 coronavirus at the structure level or in which electron microscopy photographs of the virus were presented. We restricted the survey to what we considered to be a reasonable length in terms of the time required to complete the questionnaire, deciding that the questionnaire should require no more than 35–45 minutes to complete. We calculated that a maximum of 46 images could be presented to the participants in this time and thus, those 46 images were selected by the researchers. The selection was carried out in two stages: in a first stage, we divided and quantified all the images in each of the categories (21 photographs versus 50 illustrations; 7 black and white images versus 64 colour images; 21 2D images versus 50 3D images). In a second stage, we decided that even it was not possible to offer an equal selection, the aim should be to make it as balanced as possible between the categories. After discarding three poor quality photographs, we took all of the images from the smaller categories (i.e.: 18 photographs, 7 black and white images, and 21 2D images), while we randomly selected the images from the rest of the categories (28 illustrations, 39 colour images, and 25 3D images). (See S2 Data).

### Dependent variables

Six dependent variables were selected, evoked in the participants by the images of the coronavirus used as stimuli: (1) beauty, (2) scientific quality, (3) realism, (4) degree of infectivity, (5) the level of fear, and (6) the didactic nature. These variables were chosen based on existing literature, as explained below, which were thought to be of interest in the context of the pandemic.

#### Beauty

aesthetics judgments are considered a subset of evaluative judgments, such as those made based on social, religious or moral cues [19]. However, the current pandemic is a global phenomenon where a very specific “object” is considered as an invisible, global enemy [20]. Although there are real images of SARS-CoV-2 [8], the media have published different illustrations and drawings that presumably beautify this ‘public enemy number one’ (in the words of the World Health Organization). We thought it would be interesting to know how specific formal characteristics of the coronavirus images affected the perception of beauty of this ‘public enemy number one’.

#### Scientific quality

the current pandemic (previously epidemic) has led to the presentation of scientific information at a quite unprecedented level, both among the scientific community, the media and the public at large [1]. This latter group has become vulnerable to ‘infodemics’ [21] and as such, we were interested in analysing the general public’s perception of the scientific representation of coronavirus images.

#### Realism

unfortunately, fake news is now found commonly on a daily basis in many different areas [22, 23] and the COVID-19 crisis has been no exception [24]. Due to the extended use of unrealistic images of SARS-CoV-2 in the media [5], we wanted to know the level of realism that participants attribute to the images of coronavirus.

#### Degree of infectivity

within the limited knowledge regarding the transmission of COVID-19 [2, 25, 26], especially during the first weeks of this crisis, the level of infection has been one of the most relevant topics covered by the press. For that reason, we wanted to know if specific characteristics of the visual presentation of the coronavirus could have an impact on the perception of its infectivity. This could be of great interest in a public health communication crisis.

#### Fear

according to previous studies, one of the first human reactions to a terrible pandemic is panic [27]. By deducing the level of fear that different attributes of the images can provoke in society, the media can establish a more appropriate tone for their messages.

#### Didactic nature

from their beginning, most media aim to inform, educate and entertain [28]. During this pandemic, most media channels prepare educational material about COVID-19 for their audiences [29]. Since many such media channels have used the images we presented to our participants [5], we wanted to determine the didactic value that was attributed to them.

### Independent variables

**Fig 3:**
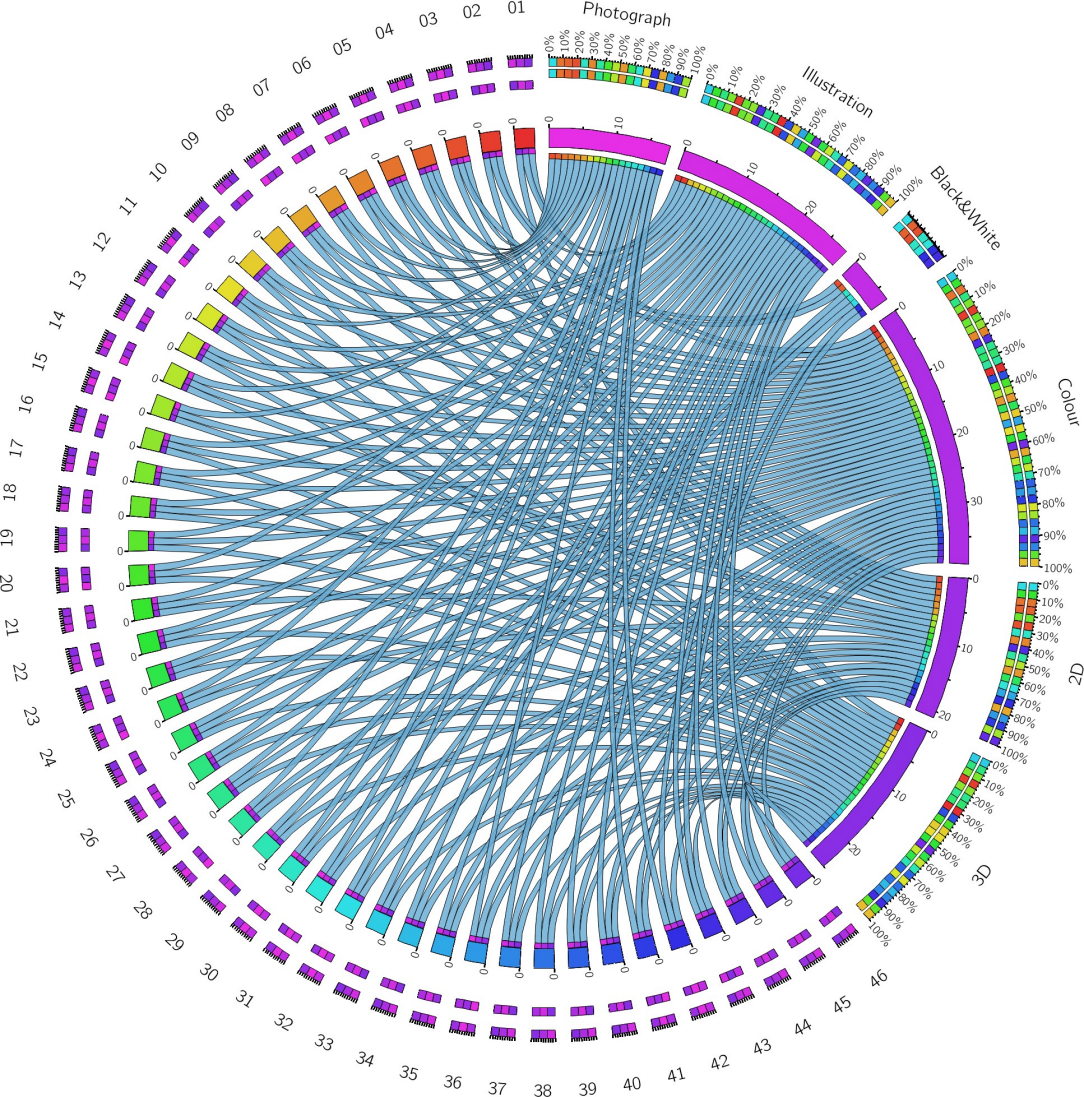
Chord diagram of the attributes of the images presented. Each number corresponds to one of the images. Each string indicates the connection between the image and its attributes, and the colours are used for ready visualization. The percentages and squares in the outer ring show the number of images needed to get a specific percentage for each attribute. Note there were fewer black and white images.

Six independent characteristics of the images were chosen to carry out this study: photograph, illustration, black and white, colour, 2D or 3D image. As mentioned, these variables were chosen based on the attributes of the stimuli used. In our sample (N = 46), there were 18 photographs, 28 illustrations, 7 black and white images, 39 colour images, 21 2D images, and 25 3D images (Fig 3).

### Colorimetry

To study the colorimetry of the images and how their colour temperature might affect their perception in terms of the categories studied, we acquired colorimetry data using a spectrometer (Sekonic C-800). We obtained the xy value to locate the colour of the images in the CIE 1931 chart. We also acquired the hue and the colour temperature in Kelvin. To obtain this data, all the images were presented in a MacBook Pro. We first calibrated the LCD display using the following chromatic parameters: red phosphor, x = 0.68, y = 0.32; green phosphor, x = 0.265, y = 0.69; blue phosphor, x = 0.151, y = 0.054; and native white point, x = 0.312, y = 0.329. Subsequently, we located the spectrometer 40 cm from the screen and presented the images individually in full screen.

### Data analysis

We obtained the mean value of the attributes analysed (photograph, illustration, colour, black and white, 2D image, 3D image) for each dependent variable (beauty, scientific nature, realism, infectivity, fear and didactic value), based on the value given by each participant to all the images with that specific attribute. We tested the normality of the data with a Shapiro-Wilk Test (see S3 Data). and we then performed 5 Friedman Test Repeated Measures ANOVA on the Rank analyses, 1 for each dependent variable. We also carried out other Friedman Tests, Repeated Measures ANOVA on Ranks, to understand how the same attribute had a different impact on the different categories. We performed Kendall’s W Test to measure effect size for Friedman Tests. We performed Pearson Product Moment Correlation Tests among the categories or dependent variables to search for correlations. With the same test, we also assessed the correlations among the categories and the colour temperature of the images. Acknowledging that one cannot theoretically guarantee the true distance between scores in the 5-point scoring system used (even though it was a scale of 1-2-3-4-5), and that the use of parametric statistics on ordinal data has been referred to as one of the “seven deadly sins of statistical analysis” [30], previous studies have already proven that parametric statistics can be applied to Likert data [31, 32].

## Results

We obtained 91,908 responses to the in-depth survey from 333 participants who assessed the perceived quality of 46 coronavirus images in terms of six categories: beauty, scientific nature, realism, infectivity, fear, and didactic nature. In total, 15,315 associations were collected for each category in the survey. Of the 333 subjects that participated in this study, around half were women (56.8%) and apart from two participants who preferred not to say, the rest were men (42.6%). There was a wide spread of the participant’s age range: 18–25 (5.1%); 26–35 (11.4%); 36–45 (20.7%); 46–55 (36.3%); 56–65 (20.7%); +65 (5.7%). Most of participants had completed University/college studies (84.7%: see Table 1 for more details).

**Table 1:** Characteristics of participants.

Characteristics	N (%)
**Gender**	
Female	189 (56.8%)
Male	142 (42.6%)
Preferred not to say	2 (0.6%)
**Age (years)**	
18–25	17 (5.1%)
26–35	38 (11.4%)
36–45	69 (20.7%)
46–55	121 (36.3%)
56–65	69 (20.7%)
+65	19 (5.7%)
**Education**	
University/college studies	282 (84.7%)
Technical training	33 (9.9%)
Secondary school education	17 (5.1%)
Primary school education	1 (0.3%)

Characteristics of participants for all three variables registered (gender, age and education).

### Beauty, scientific nature, realism, infectivity, fear and didactic nature of the SARS-CoV-2 images

**Fig 4:**
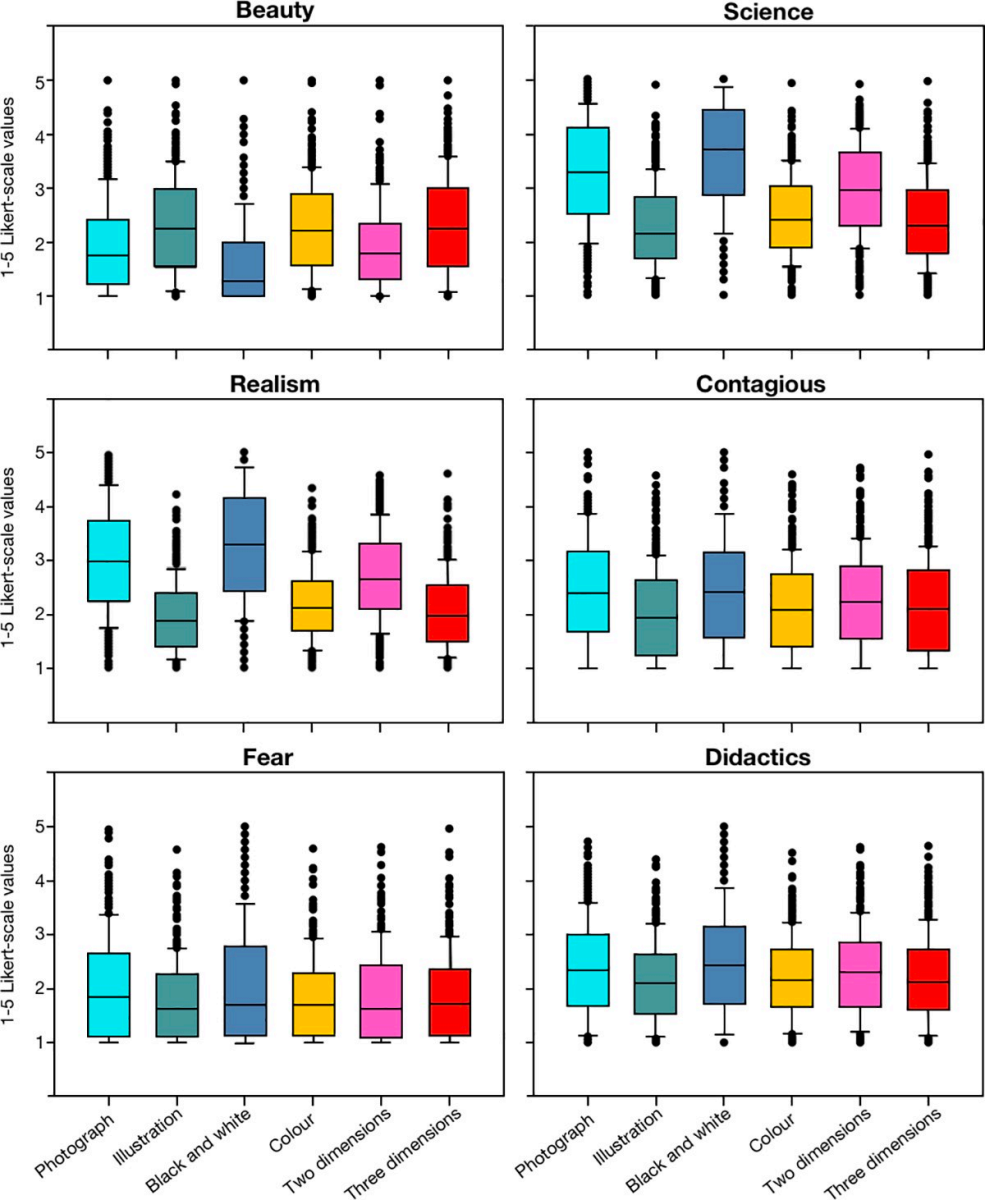
Box plot with the mean scores that each image obtained. Mean scores are distributed by category (beauty, scientific content, realism, infectivity, fear and didactic quality) and attribute (photograph, illustration, black and white, colour, two dimensions and three dimensions).

In terms of beauty, significant differences were found (Fig 4) among the aforementioned attributes (*X*^2^(5) = 610.772, *p* < 0.001, Friedman Test; *W* = 0.367, Kendall’s Test) and the median (M) value of the attributes could be ordered from highest to lowest as follows: illustration, M = 2.250; 3D, M = 2.240; colour, M = 2.205; 2D, M = 1.810; photograph, M = 1.778; black and white, M = 1.286. Accordingly, illustrations were considered the most beautiful images, and black and white images the least beautiful ones.

In terms of the scientific content of the images (*X*^2^(5) = 941.401, *p* < 0.001, Friedman Test; *W* = 0.565, Kendall’s Test), the median score of the attributes ranged from: black and white, M = 3.714; photograph, M = 3.278; 2D, M = 2.952; colour, M = 2.385; 3D, M = 2.280; illustration, M = 2.143. Hence, while the black and white images were scored as the most scientific ones, illustrations were considered to be the least scientific images.

The significant differences among the attributes in the category of realism (*X*^2^(5) = 891.015, *p* < 0.001, Friedman Test; *W* = 0.535, Kendall’s Test), were reflected by median values in the attributes from: black and white, M = 3.286; photograph, M = 2.944; 2D, M = 2.667; colour, M = 2.128; 3D, M = 1.960; illustration, M = 1.857. Thus, the fact that black and white images got the highest score in the participant’s perception of reality, while illustrations were considered the least real coincided with the category of their scientific nature. Accordingly, when participants were asked about the scientific or realistic nature of the coronavirus images, the influence of the image’s attributes on their response seemed to be quite similar.

When the participants were asked how contagious they thought the coronavirus in the images were, significant differences were again evident among the attributes (*X*^2^(5) = 359.529, *p* < 0.001, Friedman Test; *W* = 0.216, Kendall’s Test), with median values obtained from: black and white, M = 2.429; photograph, M = 2.389; 2D, M = 2.238; 3D, M = 2.080; colour, M = 2.077; illustration, M = 1.929. As such, the black and white images considered to be the most scientific and real were also thought to be the most contagious, whereas illustrations that were perceived as the most beautiful obtained the lowest score in the contagious category.

In terms of how scary an image seemed to the participants, significantly different values were associated with the attributes of the images studied (*X*^2^(5) = 164.572, *p* < 0.001, Friedman Test; *W* = 0.099, Kendall’s Test), obtaining median values ranging from: photograph, M = 1.833; 3D, M = 1.720; black and white, M = 1.714; colour, M = 1.692; 2D, M = 1.619; illustration, M = 1.607. Therefore, while photographs were thought to be the scariest, on average, the coronavirus in the images was not attributed a high score. So, in general one could say that the sample images of the coronavirus used in this study did not seem scary to the participants.

Finally, in terms of their didactic nature, the significant differences detected on the basis of the attributes studied (*X*^2^(5) = 87.868, *p* < 0.001, Friedman Test; *W* = 0.053, Kendall’s Test), were due to the median values of: black and white, M = 2.429; photograph, M = 2.333; 2D, M = 2.286; colour, M = 2.154; 3D, M = 2.120; illustration, M = 2.107. As such, black and white images were scored as the most didactics ones, while illustrations were considered the least didactic.

Although we did not design this study to assess differences based on sociodemographic characteristics, we performed multiple analyses for all three variables registered from the participants (gender, age and education). No significant differences were found in any case (see S1, S2 and S3 Tables).

### Correlations among the categories

Some interesting correlations were found among the categories for the distinct coronavirus images. As such, beautiful images were negatively correlated with a scientific perception, realism and the didactic nature of the coronavirus images, while scientific images were positively correlated with realism, infectivity, fear and the didactic nature of the coronavirus images. Similarly, realistic images were positively correlated with infection, fear and a didactic perception, while they were negatively related to beauty. Finally, contagious coronavirus images were positively correlated with a scientific quality, realism, fear and a didactic nature of the images, while scary images were positively correlated with a didactic nature, infectivity, realism and a scientific nature (see Table 2 for more details).

**Table 2:** Correlations among categories.

	Scientific	Realistic	Infectivity	Fear	Didactic
Beauty	r = - 0.423	r = - 0.446	r = - 0.140	r = - 0.079	r = - 0.316
	p = 0.003*	p = 0.002*	p = 0.353	p = 0.604	p = 0.0326*
Science		r = 0.993	r = 0.787	r = 0.641	r = 0.782
		p < 0.001**	p < 0.001**	p < 0.001**	p < 0.001**
Realism			r = 0.780	r = 0.623	r = 0.768
			p < 0.001**	p < 0.001**	p < 0.001**
Infectivity				r = 0.897	r = 0.677
				p < 0.001**	p < 0.001**
Fear					r = 0.561
					p < 0.001**

Pearson correlations among the categories of the values attributed to coronavirus images by the participants:

*p < 0.05

**p < 0.001.

### Photographs, illustrations, black and white, colour, 2D, and 3D images of SARS-CoV-2

Using the same data, we also compared the mean values associated to each attribute for every category. The aim was to understand how the same attribute had a different impact on different categories and when a Friedman repeated-measures ANOVA on ranks test was performed, significant differences were found for all the attributes. In terms of photography (*X*^2^(5) = 680.208, *p* < 0.001, Friedman Test; *W* = 0.409, Kendall’s Test) the median (M) values of the categories were: scientific, M = 3.278; realistic, M = 2.944; infectivity, M = 2.389; didactic, M = 2.333; scary, M = 1.833; and beautiful, M = 1.778. By contrast, the median values of the illustrations (*X*^2^(5) = 291.238, *p* < 0.001, Friedman Test; *W* = 0.175, Kendall’s Test) were: beauty, M = 2.250; scientific, M = 2.143; didactic, M = 2.107; contagious, M = 1.929; realistic, M = 1.857; and scary M = 1.607.

In terms of black and white images (*X*^2^(5) = 908.101, *p* < 0.001, Friedman Test; *W* = 0.545, Kendall’s Test) the median values were: scientific, M = 3.714; realistic, M = 3.286; contagious, M = 2.429; didactic, M = 2.429; scary, M = 1.714; and beautiful, M = 1.286. Whereas for colour images (*X*^2^(5) = 330.898, *p* < 0.001, Friedman Test; *W* = 0.199, Kendall’s Test), the median values obtained were: scientific, M = 2.385; beautiful, M = 2.205; didactic, M = 2.154; realistic, M = 2.128; contagious, M = 2.077; and scary, M = 1.692.

Regarding 2D images (*X*^2^(5) = 649.569, *p* < 0.001, Friedman Test; *W* = 0.390, Kendall’s Test), the median values were: scientific, M = 2.952; realistic, M = 2.667; didactic, M = 2.286; contagious, M = 2.238; beautiful, M = 1.810; and scary, M = 1.619. By contrast, the images in the 3D attribute (*X*^2^(5) = 263.637, *p* < 0.001, Friedman Test; *W* = 0.158, Kendall’s Test) obtained the following median values: scientific, M = 2.280; beautiful, M = 2.240; didactic, M = 2.120; contagious, M = 2.080; realistic, M = 1.960; and scary, M = 1.720.

### Colorimetry and colour temperature of SARS-CoV-2 images

**Fig 5:**
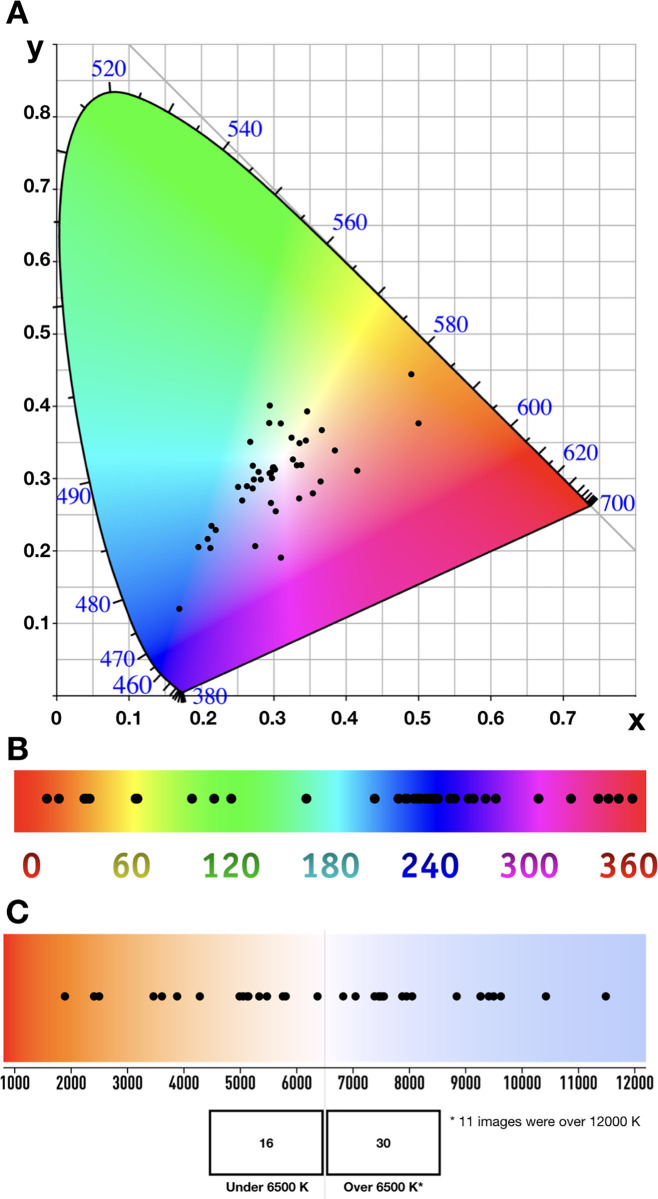
Chromaticity of the coronavirus images. Presentation of the colour parameters for each coronavirus image analysed in the different charts. In all cases, the colours shown are only a pedagogic reference approximation: A, CIE 1931 Chromaticity chart, the numbers in blue are in nanometres; B, Hue 360° Chromaticity chart with the numbers in degrees; C, Kelvin chart with the numbers are in Kelvin.

In addition, we studied the colorimetry of the images and located each one of them in a CIE 1931 chart, as well as in a Hue 360° and a Kelvin chromaticity chart (Fig 5). We found that most coronavirus images had a blue hue, while very few were within the region of a green hue. Regarding the colour temperature, we found that only 16 of the 46 coronavirus images were in the hot temperature range (below 6500 K), while the rest (65.2%) were in cold colour temperatures (above 6500 K). The colour temperature of natural daylight is approximately 6500 K, although it may vary from 6000 to 7500 K.

In the search for correlations between perception in terms of the categories studied (beauty, scientific nature, realism, infectivity, fear and didactic nature) and the colour temperature of SARS-CoV-2 images, Pearson’s correlations were obtained for the mean value given to each coronavirus image in each category and their colour temperature (in Kelvin). We failed to obtain significant correlations in most of the categories: beauty, *r* = 0.226, *p* = 0.131; scientific, *r* = -0.215, *p* = 0.151; contagious, *r* = -0.164, *p* = 0.277; fear, *r* = -0.048, *p* = 0.752; and didactic, *r* = -0.213, *p* = 0.155. However, we did detect a negative correlation in the category of realism (*r* = -0.294, *p* = 0.047), indicating that coronavirus images with a lower colour temperature had an enhanced perception of realism and vice versa.

## Discussion

Communicating scientific information to non-expert audiences should include relevant contextual information to complement the data measurements themselves [33]. Previous studies have shown that presenting different images next to the very same scientific text changes the perception and the judgement of scientific information [13]. In particular, it was found that brain images have a highly persuasive power and add credibility to the information, explaining why neuroscience has a strong presence in the media and in advertising today [34]. In this study, we analysed different attributes of images representing the SARS-CoV-2 coronavirus and the effect that these have on an individual’s perception of certain characteristics. Based on our results, the content of an image not only modifies the perception of a topic but also, so do its more formal attributes: illustration/photograph, colour/black and white, 2D/3D. This is particularly important as by modifying perception, one can clearly influence people’s behaviour [35].

Knowing how certain attributes or elements influence specific perceptions seems to be of vital importance in times of pandemics when communication can affect the behaviour of the population and society as a whole [10]. Recent studies have supported the claim that fear appeals are effective in changing health-related behaviours [36], and it has been proposed that appealing to the public through fear may be useful when combined, in a non-threatening way, with messages to improve self-efficacy and to help people change their behaviour [36]. Indeed, a linear relationship was found between fear and persuasion in a between-persons association [37].

Goethe already indicated that specific colour attributes excite particular feelings and states of mind [38]. In fact, defining the affective value of colours has long been a goal in psychology [39]. However, the Gestalt school has made important contributions to the discussion of the existence of universal artistic values that affect visual perception and that can therefore modify behaviour, not only through colour but also through form and movement [40, 41]. Communications companies have also looked for those universal perceptive features for decades, studying aspects like colour [42], image format [43] or packaging [44], among others [45, 46]. More recently, the establishment of neuroaesthetics and neuromarketing as specific disciplines further consolidates these lines of thought [47–49]. In advertising it is very clear that a consumer’s perception of a product will affect their behaviour towards the product [50, 51].

In a pharmaceutical context, the attributes of a pill can have an impact on the user’s behaviour. For example, there is evidence that green and blue colours may have more sedative effects, while red and orange are attributed more stimulant effects [52]. A recent study into scientific communication found that the type of geographic COVID-19 prevalence map used to present information affects public opinion [53]. Moreover, it has also been shown that the type of graphic accompanying news articles about medical information can influence the interest in infectious disease vaccination [54]. However, these issues do not seem to receive particular attention when preparing scientific communications or medical information. Considering that the population’s behaviour is fundamental in the current pandemic situation, scientific communication should bear these concepts in mind.

According to our results, the attributes of the images representing the SARS-CoV-2 coronavirus affect people’s perception of the virus. The beauty of coronavirus images is more likely perceived when presenting colour illustrations in 3D. From the beginning of this pandemic, most coronavirus images used were, precisely, colour 3D illustrations [5], indicating that the media was presenting beautiful images to talk about a very serious (and ugly) topic. Significantly, the real images of SARS-CoV-2 are 2D, black and white photographs, attributes that distance the individual from beauty. Note that due to the small size of SARS-CoV-2, real images of the coronavirus are black and white as they have been obtained with electron microscopy [55]. Thus, the question that arises at this point is whether or not the media should use beautiful or real images when offering information about such a serious and unpleasant topic as a pandemic.

At the same time, the photographic nature, black and white, and 2 dimensional attributes of coronavirus images enhance the perception of a more scientific, real, contagious and didactic virus, whether they are real or fake coronavirus images. Again, recent data indicates that the public has mostly been perceiving illustrated images of SARS-CoV-2 that, according to this study, do not evoke in them scientific value, reality, infectivity, fear or a highly didactic quality [5]. This seems very important due to the importance of the citizen in the strategy to fight this pandemic. Given the negative correlation found between beauty and science, communicators should also check what type of visual information they want to present to the public. Or perhaps scientists should reflect on whether scientific communication should also incorporate new pedagogic facets of scientific illustration.

It is interesting that black and white images of SARS-CoV-2 are perceived as the most realistic, scientific, contagious and didactic, which is difficult to explain given that most of us perceive reality in colour. Indeed, this seems like the classical discussion about whether we dream in colour or black and white [56–58]. However, the perception that black and white images of the coronavirus, or presumably, black and white images in general, are considered more scientific or realistic may also be related to our current perception of the truth in the media. It may even be worth also considering whether the photographs of our ancestors, since the times of daguerreotypes, might have strengthened the idea of black and white images as a criterion of reality in terms of photographic testimony. Hence, more research may be needed to determine the underlying reasons why black and white images are perceived to be more scientifically accurate and realistic.

We thought it would be interesting to study the perception of the incessant and varied images of the coronavirus that have been used globally to address the challenge of communicating information related to SARS-CoV-2 (or other future pandemics). However, we now see that more complementary studies of reception may be necessary to address this issue, such as those using neuroscientific techniques like Functional Near Infrared Spectroscopy (fNIRS) and electroencephalography (EEG). These approaches offer complementary perspectives to learn how visual information is processed in terms of visual perception and cognitive processing. Only in this way are we likely to be able to draw new and relevant conclusions about the relationship between how coronavirus images are perceived, and how this may influence an individual’s perception and behaviour.

So far, in this study we have seen how the visual presentation of SARS-CoV-2 impacts on public perception of this coronavirus. This may has had an impact on public perception of the pandemic. Our results make us think that it is possible that the public perception provoked by the visual representations of the SARS-CoV-2 may has impacted on people’s behaviour (e.g., willingness to social distance) and emotional states (e.g., anxiety). For that reason, we suggest that scientific communicators pay attention to these results for communicating science in future health contexts, in which population’s behaviour is essential.

Finally, common sense would lead us to believe that medical reporting would be accurate and complete, properly informing citizens so that they are in a position to participate and make decisions about their healthcare [12]. Could misleading images disempower people in a similar manner as misleading information does [59]? Multidisciplinary Social and Cognitive Sciences can participate in the design of communications, health programs and in the detection of disinformation, helping to understand the processes by which informative material can instil anxiety, fear and stigma [60]. Moreover, visualization is tied to aesthetic and cultural norms that should be taken into account when reproducing the appearance of health elements, like viruses [61]. Also, scientific visualization should be done bearing in mind picking colours suitable for colour-blind readers, enhancing accessibility [62]. Not only science and academic institutions but also, the Mass Media also has a great responsibility to provide correct information, appropriate headlines and when necessary, to ensure informed and serene behaviour in the general population.

## Strengths and limitations

While one positive feature of the current study is that it addresses information from the first pandemic to affect the modern, hyperconnected world, it has several limitations. Firstly, we did not calculate the sample size as the instability of the moment made it very difficult to estimate the true population available. For that reason, we estimated the participants that we wanted to answer our questionnaire but without defining an actual sample size. Also, the questionnaire was very long, so participants were required to spend considerable time completing it, which may have affected the completion rate. Indeed, another limitation of the study was that the platform used was not able to detect how many people started our questionnaire without finishing it, giving us no information about the completion rate. It was not possible to do completeness checks before submitting the questionnaire. Moreover, we have no data regarding the unique site visitors, the view rate (ratio of unique survey visitors/unique site visitors), the participation rate (ratio of unique visitors who agreed to participate/unique first survey page visitors), or the completion rate (ratio of users who finished the survey/users who agreed to participate) [63]. Another limitation is that we did not prevent multiple entries from the same individual based on cookies or IP checks, relying on a log file analysis to do so. We also did not measure the time people needed to fill in the questionnaire and we did not weight items or propensity scores [63]. Another potential limitation of our work is that most of the participants had a high level of education. This fact may have had an impact on the perception of scientific images. Future works should avoid this limitation to get broader results.

In summary, the purpose of this study was to analyse how different ways of visually representing the coronavirus that causes COVID-19 are perceived by the public. This is a little studied topic in health communication, yet it may be of potential interest for the use of visual media in health communication, and for scientific illustrators and media professionals.

## Supporting information

S1 Table Analysis of the data based on the variable gender. (PDF)

S2 Table Analysis of the data based on the variable age. (PDF)

S3 Table Analysis of the data based on the variable education. (PDF)

S1 Data Datasets with the responses of each participant to each stimulus. (XLSX)

S2 Data Characteristics of stimuli. (XLSX)

S3 Data Normality tests. (PDF)
