# Supplementary material for: The attributes of the images representing the SARS-CoV-2 coronavirus affect people’s perception of the virus
Source: PLoS One. 2021 Aug 25;16(8):e0253738. doi: 10.1371/journal.pone.0253738 (PMC8386876; doi:10.1371/journal.pone.0253738)
Supplement: S1 Table — (PDF) [file pone.0253738.s001.pdf]

S1 Table: Analysis of the data based on the gender variable. The rows show differences based on Kruskall-Wallis ANOVA on Ranks, since data did not pass normality test (Test Shapiro-Wilk).

Gender (Female, Male, Preferred not to say)	Beauty – photo	$H(2) = 1.417$, $p = 0.492$
	Beauty – illustration	$H(2) = 2.081$, $p = 0.353$
	Beauty – black and white	$H(2) = 2.084$, $p = 0.353$
	Beauty – colour	$H(2) = 0.594$, $p = 0.743$
	Beauty – 2D	$H(2) = 0.786$, $p = 0.675$
	Beauty – 3D	$H(2) = 1.453$, $p = 0.484$
	Scientific – photo	$H(2) = 1.058$, $p = 0.589$
	Scientific – illustration	$H(2) = 5.422$, $p = 0.066$
	Scientific – black and white	$H(2) = 0.00154$, $p = 0.999$
	Scientific – colour	$H(2) = 1.953$, $p = 0.377$
	Scientific – 2D	$H(2) = 1.328$, $p = 0.515$
	Scientific – 3D	$H(2) = 5.199$, $p = 0.074$
	Realism – photo	$H(2) = 0.757$, $p = 0.685$
	Realism – illustration	$H(2) = 5.063$, $p = 0.080$
	Realism – black and white	$H(2) = 0.131$, $p = 0.936$
	Realism – colour	$H(2) = 1.568$, $p = 0.457$
	Realism – 2D	$H(2) = 0.504$, $p = 0.777$
	Realism – 3D	$H(2) = 4.431$, $p = 0.109$
	Contagious – photo	$H(2) = 8.119$, $p = 0.017$*
	Contagious – illustration	$H(2) = 3.541$, $p = 0.170$
	Contagious – black and white	$H(2) = 9.207$, $p = 0.010$*
	Contagious – colour	$H(2) = 4.650$, $p = 0.098$
	Contagious – 2D	$H(2) = 8.950$, $p = 0.011$*
	Contagious – 3D	$H(2) = 3.308$, $p = 0.191$
	Scary – photo	$H(2) = 7.534$, $p = 0.023$*
	Scary – illustration	$H(2) = 4.586$, $p = 0.101$
	Scary – black and white	$H(2) = 8.161$, $p = 0.017$*
	Scary – colour	$H(2) = 5.460$, $p = 0.065$
	Scary – 2D	$H(2) = 8.396$, $p = 0.015$*
	Scary – 3D	$H(2) = 4.239$, $p = 0.120$
	Didactic – photo	$H(2) = 3.649$, $p = 0.161$
	Didactic – illustration	$H(2) = 4.073$, $p = 0.131$
	Didactic – black and white	$H(2) = 1.834$, $p = 0.400$
	Didactic – colour	$H(2) = 3.026$, $p = 0.220$
	Didactic – 2D	$H(2) = 4.235$, $p = 0.120$
	Didactic – 3D	$H(2) = 3.257$, $p = 0.196$

* After computing all pairwise multiple comparison procedures (Dunn's Method), to isolate the group that differ from the others, we found that the group was "Preferred not to say". Since this group was formed by just 2 participants, these results are not understood as relevant for our study. No significant differences were found between female and male groups.
