# Supplementary material for: The attributes of the images representing the SARS-CoV-2 coronavirus affect people’s perception of the virus
Source: PLoS One. 2021 Aug 25;16(8):e0253738. doi: 10.1371/journal.pone.0253738 (PMC8386876; doi:10.1371/journal.pone.0253738)
Supplement: S2 Table — (PDF) [file pone.0253738.s002.pdf]

S2 Table: Analysis of the data based on the age variable. The rows show differences based on Kruskall-Wallis ANOVA on Ranks, since data did not pass normality test (Test Shapiro-Wilk).

Age (years) 18-25 26-35 36-45 46-55 56-65 +65	Beauty – photo	$H(5) = 5.330, p = 0.377$
	Beauty – illustration	$H(5) = 5.168, p = 0.396$
	Beauty – black and white	$H(5) = 10.146, p = 0.071$
	Beauty – colour	$H(5) = 3.853, p = 0.571$
	Beauty – 2D	$H(5) = 5.291, p = 0.381$
	Beauty – 3D	$H(5) = 5.373, p = 0.372$
	Scientific – photo	$H(5) = 6.414, p = 0.268$
	Scientific – illustration	$H(5) = 1.766, p = 0.881$
	Scientific – black and white	$H(5) = 5.478, p = 0.360$
	Scientific – colour	$H(5) = 2.325, p = 0.803$
	Scientific – 2D	$H(5) = 5.483, p = 0.360$
	Scientific – 3D	$H(5) = 1.948, p = 0.856$
	Realism – photo	$H(5) = 8.063, p = 0.153$
	Realism – illustration	$H(5) = 1.442, p = 0.920$
	Realism – black and white	$H(5) = 6.503, p = 0.260$
	Realism – colour	$H(5) = 3.795, p = 0.579$
	Realism – 2D	$H(5) = 8.320, p = 0.139$
	Realism – 3D	$H(5) = 1.284, p = 0.937$
	Contagious – photo	$H(5) = 7.906, p = 0.161$
	Contagious – illustration	$H(5) = 6.798, p = 0.236$
	Contagious – black and white	$H(5) = 10.169, p = 0.071$
	Contagious – colour	$H(5) = 7.855, p = 0.164$
	Contagious – 2D	$H(5) = 8.268, p = 0.142$
	Contagious – 3D	$H(5) = 7.861, p = 0.164$
	Scary – photo	$H(5) = 8.175, p = 0.147$
	Scary – illustration	$H(5) = 7.325, p = 0.198$
	Scary – black and white	$H(5) = 7.840, p = 0.165$
	Scary – colour	$H(5) = 7.837, p = 0.165$
	Scary – 2D	$H(5) = 8.127, p = 0.149$
	Scary – 3D	$H(5) = 7.504, p = 0.186$
	Didactic – photo	$H(5) = 4.496, p = 0.480$
	Didactic – illustration	$H(5) = 3.370, p = 0.643$
	Didactic – black and white	$H(5) = 6.350, p = 0.274$
	Didactic – colour	$H(5) = 3.732, p = 0.589$
	Didactic – 2D	$H(5) = 4.834, p = 0.436$
	Didactic – 3D	$H(5) = 3.674, p = 0.597$
