# Supplementary material for: The attributes of the images representing the SARS-CoV-2 coronavirus affect people’s perception of the virus
Source: PLoS One. 2021 Aug 25;16(8):e0253738. doi: 10.1371/journal.pone.0253738 (PMC8386876; doi:10.1371/journal.pone.0253738)
Supplement: S3 Data — (PDF) [file pone.0253738.s006.pdf]

Normality Test (Shapiro-Wilk)

Data source: Data

Beauty_Photograph:	W-Statistic = 0,901	P < 0,001	Failed
Beauty_Illustration:	W-Statistic = 0,963	P < 0,001	Failed
Beauty_Blackandwhite:	W-Statistic = 0,758	P < 0,001	Failed
Beauty_Colour:	W-Statistic = 0,968	P < 0,001	Failed
Beauty_2D:	W-Statistic = 0,921	P < 0,001	Failed
Beauty_3D:	W-Statistic = 0,964	P < 0,001	Failed
Scientific_Photograph:	W-Statistic = 0,976	P < 0,001	Failed
Scientific_Illustration:	W-Statistic = 0,967	P < 0,001	Failed
Scientific_Blackandwhite:	W-Statistic = 0,956	P < 0,001	Failed
Scientific_Colour:	W-Statistic = 0,985	P = 0,002	Failed
Scientific_2D:	W-Statistic = 0,986	P = 0,002	Failed
Scientific_3D:	W-Statistic = 0,975	P < 0,001	Failed
Realism_Photograph:	W-Statistic = 0,981	P < 0,001	Failed
Realism_Illustration:	W-Statistic = 0,944	P < 0,001	Failed
Realism_Blackandwhite:	W-Statistic = 0,967	P < 0,001	Failed
Realism_Colour:	W-Statistic = 0,977	P < 0,001	Failed
Realism_2D:	W-Statistic = 0,986	P = 0,003	Failed
Realism_3D:	W-Statistic = 0,959	P < 0,001	Failed
Infectivity_Photograph:	W-Statistic = 0,959	P < 0,001	Failed
Infectivity_Illustration:	W-Statistic = 0,936	P < 0,001	Failed
Infectivity_Blackandwhite:	W-Statistic = 0,952	P < 0,001	Failed
Infectivity_Colour:	W-Statistic = 0,953	P < 0,001	Failed
Infectivity_2D:	W-Statistic = 0,958	P < 0,001	Failed
Infectivity_3D:	W-Statistic = 0,945	P < 0,001	Failed
Fear_Photograph:	W-Statistic = 0,896	P < 0,001	Failed
Fear_Illustration:	W-Statistic = 0,897	P < 0,001	Failed
Fear_Blackandwhite:	W-Statistic = 0,882	P < 0,001	Failed
Fear_Colour:	W-Statistic = 0,901	P < 0,001	Failed
Fear_2D:	W-Statistic = 0,887	P < 0,001	Failed
Fear_3D:	W-Statistic = 0,908	P < 0,001	Failed
Didactic_Photograph:	W-Statistic = 0,971	P < 0,001	Failed
Didactic_Illustration:	W-Statistic = 0,970	P < 0,001	Failed
Didactic_Blackandwhite:	W-Statistic = 0,967	P < 0,001	Failed
Didactic_Colour:	W-Statistic = 0,978	P < 0,001	Failed
Didactic_2D:	W-Statistic = 0,978	P < 0,001	Failed
Didactic_3D:	W-Statistic = 0,970	P < 0,001	Failed

A test that fails indicates that the data varies significantly from the pattern expected if the data was drawn from a population with a normal distribution.
A test that passes indicates that the data matches the pattern expected if the data was drawn from a population with a normal distribution.
